# Acute Caffeine Supplementation Does Not Improve Performance in Trained CrossFit^®^ Athletes

**DOI:** 10.3390/sports8040054

**Published:** 2020-04-23

**Authors:** Jesse A. Stein, Melitza Ramirez, Katie M. Heinrich

**Affiliations:** Department of Kinesiology, College of Health and Human Sciences, Kansas State University, Manhattan, KS 66506, USA; melitzar@ksu.edu (M.R.); kmhphd@ksu.edu (K.M.H.)

**Keywords:** high intensity functional training, exercise, muscular endurance, ergogenic aids, sports nutrition

## Abstract

Caffeine’s ergogenic effects persist during various exercise modalities; however, information establishing its efficacy during CrossFit^®^ protocols is limited. This study aimed to determine the effects of caffeine supplementation on CrossFit^®^ performance. Twenty CrossFit^®^-trained men (age = 26.7 ± 6.2 years, experience = 3.7 ± 2.9 years) were randomized in a double-blind, crossover design. Participants completed two sessions separated by a seven-day washout period, 60 min after consuming 5 mg/kg body mass of caffeine or a placebo. In each session, participants completed as many rounds as possible in 20 min of five pull-ups, 10 push-ups, and 15 air squats. CrossFit^®^ performance was the total number of repetitions completed in 20 min. Paired-samples t-tests were used to compare CrossFit^®^ performance between caffeine and placebo conditions and to test for a potential learning effect between the first and second sessions. CrossFit^®^ performance was not significantly different during the caffeine condition compared to the placebo (468.6 ± 114.7 vs. 466.7 ± 94.3 repetitions, *p* = 0.861). A significant learning effect was identified between the first and second sessions (452.4 ± 101 vs. 483.8 ± 106.5 repetitions, *p* = 0.001), with no significant effect of treatment order (*p* = 0.438). Caffeine’s ergogenic effect were not present during the CrossFit^®^ workout “Cindy”; however, future research should include familiarization sessions and examine other CrossFit^®^ workouts in novice and women participants.

## 1. Introduction

Caffeine supplementation is pervasive in sporting disciplines, with up to 74% of Spanish elite athletes consuming caffeine prior to competition for its ergogenic effects [1]. Support for this strategy is recognized by the International Olympic Committee and the International Society of Sports Nutrition, who both acknowledge caffeine as a dietary supplement with “good evidence” for its ergogenic effects, benefiting endurance and strength/power in athletes [2,3]. The majority of this evidence has been established utilizing singular exercise modalities when assessing muscle function (muscular strength and endurance) or exhaustive protocols (time-to-exhaustion and repeated-sprint ability) [4,5,6,7,8]. However, some athletes are not easily classified into physical demands that are strictly endurance- or strength/power-based in nature and require multiple facets of health- (e.g., aerobic capacity and muscular strength) and skill-related (e.g., agility, speed, and power) physical fitness [9]. Recently, Mielgo-Ayuso addressed this concern within soccer players; however, a majority of the reviewed investigations evaluated aspects of physical performance (i.e., speed, power, agility, and time-to-exhaustion) with limited data reported regarding simulated performance [10]. Reasonably, instructing athletes with diverse physical demands to consume caffeine for performance benefits has limited evidence to support its efficacy [10,11]. Since preserving muscle function during competition is important for preventing premature fatigue, investigating the role of acute caffeine supplementation during combined exercise modalities is warranted.

The mechanisms of caffeine’s physiological effects have spanned decades of research, which has offered multiple theories for caffeine’s ergogenic properties [7]. Caffeine—structurally similar to the neuromodulator adenosine that accumulates during periods of high adenosine triphosphate (ATP) breakdown (i.e., exercise)—is an adenosine receptor antagonist that acts on multiple tissues [7,12]. The adenosine receptors have genetic polymorphisms that may predispose to caffeine consumption habits and put some individuals at risk of the negative side-effects from caffeine consumption, such as increases in anxiety and gastrointestinal distress when consumed beyond moderate doses (>6 mg·kg^−1^ body mass) [12]. Caffeine was originally believed to promote glycogen sparing during exercise by the catecholamine-induced mobilization of free fatty acids; however, augmentations of ATP provision from altered metabolism have minimal support [7]. To date, it is understood that adenosine receptor antagonism via caffeine supplementation influences both central and peripheral aspects of human physiology and perceptual responses to exercise that contribute to caffeine’s ergogenic properties [12]. These mechanisms of action include reduced pain and perceived exertion, the stimulation of the ventilatory response, and changes in the muscle milieu by the retention of potassium ions and increased release of calcium ions from the sarcoplasmic reticulum [7,12]. Emerging evidence suggests that caffeine may stimulate increases in blood flow and muscle tissue saturation via endothelial nitric oxide synthase activity [13,14]. Increases in blood flow and O_2_ availability are reported to increase muscle function by enhancing the metabolic steady-state of muscle tissue during both upper and lower-extremity exercise performed until failure [15,16].

In a recent meta-analysis of the effects of caffeine supplementation on muscle function, the authors reported significant improvements (+6–7%) in muscular endurance after caffeine supplementation [17]. A majority of the 17 investigations cited in the review assessed large muscle group(s) muscular endurance via repetitions to failure over the course of multiple weightlifting sets separated by recovery periods [18,19,20,21]. These investigations utilized isotonic exercise machines and reported relatively low repetitions (<30) even over the course of multiple sets, which may not fully translate to athletic performance.

The recent dramatic increase in high-intensity functional training—which aims to enhance multiple domains of physical fitness by temporally exposing athletes to varying modes of exercise (e.g., endurance and resistance) within and between each session, for varying durations (e.g., 2–60 min), at a relatively high-intensity [22]—has been primarily driven by CrossFit^®^, which has an annual competition called the CrossFit^®^ Games [22,23,24,25,26]. Within the athletic performance environment of CrossFit^®^, repetitions can approach up to 700 in a 20-min training session [25,27]. Similar to other sports, CrossFit^®^ athletes likely possess high levels of health- and skill-related aspects of physical fitness, and research has significantly correlated CrossFit^®^ performance with aerobic capacity, muscular strength (upper- and lower-body), and power [24,27,28]. Unfortunately, limited evidence from the sports nutrition community exists regarding the utility of dietary supplementation for CrossFit^®^ performance [29].

The current evidence examining the effects of dietary supplementation on CrossFit^®^ performance is limited, with only one investigation examining the effect of acute caffeine supplementation on CrossFit^®^ performance [30]. The recent investigation by Fogaca and colleagues found no significant effect of acute caffeine supplementation on CrossFit^®^ performance; however, their investigation may be limited by the small sample size (n = 9), CrossFit^®^ performance that included power-based exercises (snatches and double-unders), and short duration of the CrossFit^®^ workout (10 min). These limitations may be resolved with larger sample sizes, using exercises that tax other aspects of muscular fitness, and greater workout durations, the latter of which may reveal caffeine’s ergogenic properties [31,32]. Ostensibly, caffeine represents an ideal candidate for investigation, with “good evidence” establishing its ergogenicity across a variety of exercise protocols (e.g., endurance, high-intensity, muscular endurance, sprint performance, and maximal strength) and muscle groups [5,31,33]. However, the effects of caffeine supplementation on performance during high-volume muscular endurance workouts that tax multiple muscle groups with limited recovery remains unknown. In this study, the effects of caffeine supplementation on CrossFit^®^ performance for a 20-min muscular endurance workout (“Cindy”) were examined. Similar to previous investigations documenting caffeine’s ergogenic effects for other types of training programs [5,31,33], it was hypothesized that caffeine supplementation would result in an increase in CrossFit^®^ performance during a high-volume muscular endurance workout that taxed multiple muscle groups with limited recovery, as well as a decrease in perceptual responses to exercise (i.e., perceived exertion). 

## 2. Materials and Methods

### 2.1. Design and Participants

This study used a randomized, double-blind, crossover design to determine the effects of caffeine on CrossFit^®^ performance. Inclusion criteria were having ≥6 months of CrossFit^®^ experience; previously completing “Cindy” or a workout with similar repetition volumes for the included movements; being of male sex; and being 18–45 years of age. Participants were excluded if they had any known health-problem (e.g., physical and mental), answered “Yes” to any physical activity readiness questionnaire items, reported allergies or negative side-effects with caffeine use, were unable to perform “Cindy” as prescribed, or were taking medication for seizures. An a priori power analysis was conducted using a large effect size, powered at 80%, and an α of 0.05 [6]. The a priori power analysis revealed that at least 15 subjects were needed for the study to be adequately powered for statistical analysis. This research project was approved by Kansas State University’s Institutional Review Board (#9100). All subjects completed a brief online survey to determine eligibility and provided written informed consent for the study in person.

### 2.2. Measures and Procedures

Participants were asked to refrain from caffeine, alcohol, vigorous exercise, and nicotine for 24 h, maintain their normal diet, adequately hydrate, refrain from eating 3 h prior to testing, and otherwise maintain their usual training regimen throughout the study. Anthropometric measures were taken on the first laboratory visit. Height was measured using a stadiometer. Body mass and percent body fat were determined using bioelectrical impedance analysis in standard mode (TBF-300A; Tanita, Japan). Daily caffeine consumption was determined using a 7-day caffeine recall [34]. Following anthropometric measures, participants (n = 20, age = 26.7 ± 6.2 years, height = 178.6 ± 4.8 cm, mass = 84.0 ± 9.7 kg, body mass index = 26.3 ± 2.5 kg/m^2^, percent body fat = 19.3% ± 3.4%, experience = 3.7 ± 2.9 years, daily caffeine intake = 288.3 ± 287.5 mg/day) were randomized to consume either caffeine or the placebo. To determine the treatment order (placebo then caffeine vs. caffeine then placebo), participants were randomized using a random number generator (0–99), with odd numbers being placed into the placebo condition and even numbers being placed into the caffeine condition for the first visit (placebo then caffeine: n = 10; caffeine then placebo: n = 10). The caffeine pill(s) (Prolab) was/were provided at 5 mg·kg^−1^ body mass [35]. (A) 300 μg biotin pill(s) was/were used as a placebo to match for the color and texture of caffeine pills. The same amount of pill(s) of biotin were administered as the caffeine pill(s) to ensure blinding. Participants began a self-selected warm-up 50 min after the consumption of the pill(s). Participants began the CrossFit^®^ workout—“Cindy”—60 min after the consumption of the pill(s). Sixty min was selected based on caffeine’s pharmacokinetics and on investigations evaluating the effects of acute caffeine supplementation [11,36]. The workout was performed indoors in a gym, with the environmental temperature set to 22 °C (22.2 ± 0.4 °C). Each participant performed the workout alone, with no clock or timing device visible to them and without music. “Cindy” was chosen since it is a standardized CrossFit^®^ workout, and it has been previously described in the literature [25,26,37]. Briefly, participants completed as many rounds as possible of five pull-ups, 10 push-ups, and 15 air squats, in 20 min. CrossFit^®^ movement standards were followed; kipping was allowed for the pull-ups; push-ups were performed on the toes, with the subject lowering himself with a straight body until his chest touched the floor; and air squats required subjects to reach full knee and hip extension at the top of each repetition and have their hip crease below their knee at the bottom of each repetition [37]. Judges, with CrossFit^®^ Level 1 or 2 Certificates, verbally counted repetitions. Repetitions that did not meet movement standards were not counted, and participants were provided with feedback to meet the movement standards. CrossFit^®^ performance was the total number of repetitions completed in 20 min. Participants were given a post-exercise survey to determine their ratings of perceived exertion (RPE) achieved during the workout on a scale of 1–10, and if they perceived an effect from the supplement given (yes/no) [38]. Participants returned to the laboratory after a 7-day washout period, consumed the pill(s) opposite to that/those from their first visit (placebo n = 10; caffeine n = 10), and identical procedures were followed for the workout and post-workout survey. Participants were sent an electronic survey after each testing session to document any side effects from the supplement provided. For each participant, the testing sessions were scheduled within a 2 h window to avoid the caffeine performance variability associated with circadian rhythms previously described [39,40]. The percent change in the performance (i.e., repetitions completed) was calculated to assess the individual effects of caffeine supplementation ((caffeine − placebo)/(placebo) × 100) and the learning effect ((session 2 − session 1)/(session 1) × 100). 

### 2.3. Analysis

Data were entered into SPSS 25 (IBM, Armonk, NY, USA) for analysis. Dependent variables were tested for normality using the Kolmogorov-Smirnov test. The CrossFit^®^ performance variables were normally distributed and were described as mean and standard deviation. RPE was not normally distributed and was analyzed with a Wilcoxon Signed Rank test and described as median and standard deviation. Paired samples t-tests were used to determine differences between the caffeine and placebo conditions in the total numbers of repetitions. An additional paired samples t-test was used to determine if a learning effect was present between the first and second sessions. An analysis of covariance was used to determine differences in the total number of repetitions performed during the caffeine condition between the different treatment order groups (placebo–caffeine vs. caffeine–placebo), while controlling for the total number of repetitions performed during the placebo condition. Descriptive data are provided as mean ± standard deviation. The degrees of freedom (df), critical value (cv), level of significance (p), and effect size (ES) were reported for each paired samples t-test (t). The magnitude to treatment effects (ES) were estimated with Cohen’s D and classified as “trivial” (<0.19), “small” (0.20–0.49), “moderate” (0.50–0.79), and “large” (>0.80) [41]. A significance level was set at *p* < 0.05.

## 3. Results

**Figure 1:**
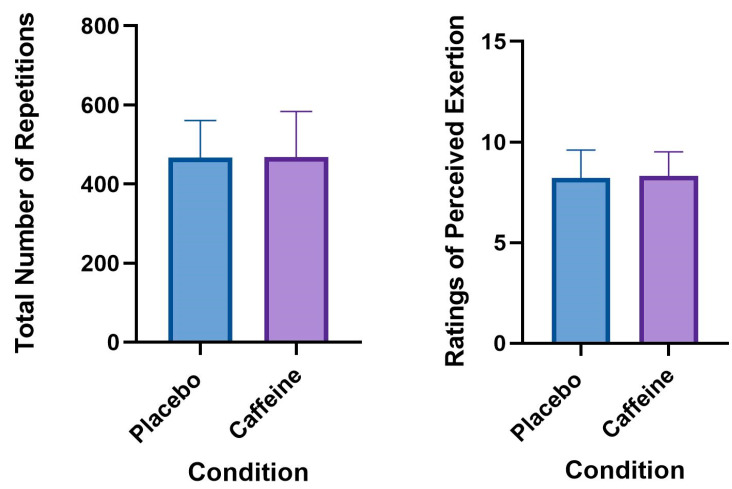
(**Left**) Performance (i.e., repetitions performed) in the caffeine and placebo conditions. Data represent the mean ± SD. (**Right**) Perceptual responses to exercise (i.e., ratings of perceived exertion (RPE)) in the caffeine and placebo conditions. Data represent the median ± SD.

No significant differences were found for CrossFit^®^ performance (i.e., the total number of repetitions performed) between the caffeine (468.6 ± 114.7 repetitions) and placebo conditions (466.7 ± 94.3 repetitions; *t*(19) = −0.178, *p* = 0.861) (Figure 1). No significant differences were found for the median perceptual exercise response ranks (i.e., RPE) between the caffeine (8.3 ± 0.3) and placebo conditions (8.2 ± 0.3, *Z*= −0.471, *p* = 0.637) (Figure 1). A significant learning effect was identified between the first and second sessions; 452.4 ± 101 vs. 483 ± 106.5 repetitions, *t*(19) = −3.791, *p* = 0.001, ES = 0.28 (0.09 0.50). After controlling for the total number of repetitions performed during the placebo condition, no significant treatment order effect was observed for the total number of repetitions performed during the caffeine condition between the different treatment order groups; F(1,20) = 0.632, *p* = 0.438. Table 1 shows the percent change in performance (i.e., total repetitions) ((caffeine − placebo)/(placebo) × 100) performed across all participants (0.19% (−4.5%, 4.9%)) and presents the learning effect and shows the percent change in performance (i.e., total repetitions) ((session 2 − session 1)/(session 1) × 100) between sessions across all participants (7.3% (3.5%, 11.1%)).

Ten of the 20 subjects (50%) perceived an effect of the supplement during the caffeine condition, while seven subjects (35%) perceived an effect of the supplement during the placebo condition. After the caffeine condition, participants reported nervousness (n = 1), gastrointestinal problems (n = 1), headache (n = 1), and increased activeness (n = 6). After the placebo condition, some participants reported nervousness (n = 2), gastrointestinal problems (n = 3), headaches (n = 1), and increased activeness (n = 2).

## 4. Discussion

The purpose of this investigation was to determine the effects of acute caffeine supplementation on CrossFit^®^ performance and perceptual responses to exercise in CrossFit^®^-trained men. Because of the well-documented ergogenic effects of caffeine that are likely due to central mechanisms [2,3,5,42], it was hypothesized that caffeine supplementation would improve CrossFit^®^ performance. The hypothesis was not supported, as no significant improvement in CrossFit^®^ performance or perceptual responses was found after caffeine supplementation. However, a significant learning effect between the first and second sessions of “Cindy” was found. Lastly, this study aimed to provide a novel contribution to the literature regarding caffeine supplementation and muscular endurance by providing a unique high-volume muscular endurance challenge. On average, participants in the current investigation performed over 400 repetitions for the 20 min workout during the caffeine and placebo conditions, which adequately addressed this challenge. To the best of the authors’ knowledge, this is the first study to determine the effects of caffeine supplementation on performance during the CrossFit^®^ workout “Cindy”, which taxes multiple muscle groups for an extended period of time with minimal rest.

In the current study, caffeine supplementation (5 mg·kg^−1^ body mass) did not significantly increase the mean CrossFit^®^ performance. These results echo the findings of Fogaca and colleagues, who found no effect of a 6 mg·kg^−1^ body mass dose of caffeine on CrossFit^®^ performance prior to a CrossFit^®^ bout including as many rounds as possible of double-unders and power snatches in 10 min [30]. Caffeine’s ergogenic effect has been reported to improve performance by 5.5–8.5% during other repeated-high-intensity efforts in team sports athletes, and by 6–7% during muscular endurance exercise [4,17,43]. Similar to these findings, some investigations have failed to find a significant effect on muscular endurance after caffeine supplementation [18,44,45]. Previous investigations determining the effects of caffeine supplementation on muscular endurance had participants perform a comparatively low number of repetitions (<30) until failure over numerous sets (≥3), separated by rest periods, and these were usually within isolated muscle groups [16,17,35].

The current investigation provides a unique addition to the literature, as participants were instructed to complete as much work as possible within the 20 min time limit while performing multiple multi-joint body weight exercises. Multi-joint exercises have been speculated to increase RPE [5]; however, similar to some investigations, significant changes in RPE between the caffeine and placebo conditions were not detected [10,20,46]. However, other investigations assessing the effects of caffeine on RPE during resistance training exercise have provided mixed results [5]. Thus, researchers might consider additional measurements of during-workout exertion, since RPE is usually taken after the exercise bout, given the nature of resistance exercise [47].

The current investigation assessed CrossFit^®^ performance via “Cindy”, which has been the most studied CrossFit^®^ workout to date [25,26,37]. Butcher and colleagues reported “Cindy” performance between competitive, experienced, and novice CrossFit^®^ athletes completing 698, 469, and 389 repetitions, respectively [25,27]. The current study, which aimed to recruit CrossFit^®^-trained (i.e., experienced) participants, seems to follow those trends regarding the numbers of repetitions performed. The significant learning effect observed appears to echo findings by Crawford and associates [28], who measured work capacity derived from performance during a similar CrossFit^®^ workout and reported a ~16% increase in work capacity after 6 weeks of a high-intensity functional training intervention that followed a CrossFit^®^ template for novice healthy adult participants. 

While the intervention by Crawford did elicit improvements in physical fitness (range 3.3–8.8%), it is possible that the increase in work capacity reported could be due to a learning effect, as the investigators did not familiarize subjects with the work capacity test [28]. Moreover, some authors have urged at least two familiarization sessions when using protocols that subjects are unaccustomed to in order to reduce the influence of systematic error [48]. This study attempted to limit this error by asking participants if they had completed “Cindy” or a similar workout. Although a small effect size for this learning effect was found, a 7.3% improvement in CrossFit^®^ performance would likely pose a meaningful advantage during competition. Collectively, these findings may be a concern for researchers and practitioners, as the learning effect during CrossFit^®^-based workouts may outweigh the effects of ergogenic aids, even among trained CrossFit^®^ participants, where the learning effect is thought to be low. 

The strengths of the current investigation include a robust study design, with subjects serving as their own controls, the recruitment of CrossFit^®^-trained men who were able to complete “Cindy” as prescribed as a high-volume muscular endurance workout, and the fact that it is the first study to document a learning effect during a CrossFit^®^ workout. However, the current investigation does not escape limitations. The significant learning effect documented may have been absolved with the inclusion of a familiarization session; however, efforts were made to reduce the participant burden with just two laboratory visits. Although 20 CrossFit^®^-trained men completed the study, the sample size is reasonably small. However, the average sample size reported in a recently published systematic review on the effects of caffeine in trained soccer players was 15 participants; therefore, the current investigation reflects similar sample sizes compared to other studies regarding caffeine’s ergogenic effect in trained populations [10]. 

Participant training volume was not reported leading up to or during the investigation. Although participants were instructed not to exercise vigorously for 24 h prior to each testing session or change their training regimen during the study period, fatigue and/or delayed onset muscle soreness from other training sessions could impact the results. However, fatigue and/or delayed onset muscle soreness was unlikely given the significant increase in performance during the second session. Additionally, invasive measures to determine blood caffeine concentration and caffeine metabolism were not evaluated. As recently identified, caffeine supplementation may have a “responder” vs. “non-responder” nature, which limits the translation to “non-responder” populations [33]. The current investigation found a significant learning effect; however, it is possible that participants 2, 3, 8, 9, 10, 11, 12, 15, 17, 18, 19, and 20 may have been “non-responders” to caffeine supplementation (see Table 1). The inter-individual differences in the ergogenicity of caffeine are thought to be related to genetic polymorphisms associated with the CYP1A2 and ADORA2A genes, which discern fast and slow caffeine metabolism/clearance [49,50]. The CYP1A2 gene, which codes for enzymes responsible for 95% of caffeine metabolism, is associated with “non-responders” to caffeine supplementation and is carried by 46% of the general population, a proportion similar to the 60% of “non-responders” in the current investigation. Although the current investigation did not characterize the genetic differences among participants, these differences may explain the “responder” vs. “non-responder” nature of the findings and presents an avenue for future investigations. However, to truly elucidate “responders” vs. “non-responders”, a baseline control condition, where no supplements are provided to the participants prior to the exercise bout, is necessary. 

Additionally, the effects of caffeine supplementation were evaluated in men and may not be generalizable to women CrossFit^®^ participants. Caffeine supplementation in women is complicated by the effects of estrogen and oral contraceptive steroids on caffeine metabolism, both of which appear to prolong the effects of caffeine in the body [5,51]. To increase internal validity, participants performed the workout alone, with no clock visible, and no music was playing. Results may differ when “Cindy” is performed in a group setting with a visible clock and music playing [52,53,54]. This investigation utilized a 10-point Likert scale for RPE and may not be sensitive enough to capture perceptual changes during CrossFit^®^ protocols. These results also reflect the findings of Fogaca and colleagues who reported no difference in post-workout RPE between the caffeine and placebo conditions using the CR10 Borg Scale [30]. Although mixed results exist for 10-point and 15-point scales for RPE during caffeine supplementation utilizing resistance- and endurance-based protocols, a recent publication by Crawford and colleagues highlights that a 15-point scale may be more appropriate in CrossFit^®^ athletes [5,47,55]. Lastly, this study utilized a dichotomous questionnaire to determine the perceived effect of the supplement, which did not capture non-decisive responses (i.e., “don’t know” or “not sure”). Because of this, the effects of supplement identification cannot be explored, which may improve performance when participants correctly identify the supplement with an active ingredient or incorrectly identify the placebo [56]. However, 50% of the participants in the current investigation perceived an effect from the supplement in the caffeine condition (i.e., correct identification), which failed to produce a significant effect on CrossFit^®^ performance.

## 5. Conclusions

In conclusion, caffeine supplementation at 5 mg·kg^−1^ body mass ingested 60 min prior to the CrossFit^®^ workout “Cindy” did not change the number of repetitions performed in CrossFit^®^-trained men. Additionally, despite the central effects of caffeine, no significant changes in perceptual responses during the exercise bout were found. Caffeine’s ergogenic effects are well documented in the literature but were not present during the CrossFit^®^ workout “Cindy”. Practitioners and athletes should be aware that a learning effect with CrossFit^®^ workouts seems likely and that some caffeine “non-responders” may exist; thus, evaluating caffeine’s effectiveness prior to competition for a specific athlete is encouraged.

## Figures and Tables

**Table 1:** Percent changes in performance between sessions (i.e., learning effect) and conditions. Percent changes between sessions represent ((session 2 − session 1)/(session 1) × 100). Percent changes between conditions represent ((caffeine − placebo)/(placebo) × 100).

Subject ID	Session 1 Treatment	Session 1 Total Repetitions	Session 2 Total Repetitions	Percent Change between Sessions	Percent Change between Conditions
1	Placebo	214	240	12.1	12.1
2	Caffeine	300	339	13.0	−11.5
3	Caffeine	372	373	0.3	−0.3
4	Placebo	581	611	5.2	5.2
5	Placebo	458	485	5.9	5.9
6	Placebo	431	456	5.8	5.8
7	Placebo	510	611	19.8	19.8
8	Caffeine	467	512	9.6	−8.8
9	Caffeine	461	486	5.4	−5.1
10	Caffeine	374	473	2.0	−20.9
11	Placebo	460	458	−0.4	−0.4
12	Placebo	423	421	−0.5	−0.5
13	Caffeine	533	474	−11.1	12.4
14	Placebo	519	551	6.2	6.2
15	Caffeine	585	607	3.8	−3.6
16	Placebo	534	541	1.3	1.3
17	Caffeine	379	410	3.8	−7.6
18	Placebo	630	711	8.2	12.9
19	Caffeine	366	420	14.8	−12.9
20	Caffeine	450	480	6.7	−6.3

## References

[1] Del Coso J., Muñoz G., Muñoz-Guerra J. (2011). Prevalence of caffeine use in elite athletes following its removal from the World Anti-Doping Agency list of banned substances. Appl. Physiol. Nutr. Metab..

[2] Maughan R.J., Burke L.M., Dvorak J., Larson-Meyer D.E., Peeling P., Phillips S.M., Rawson E.S., Walsh N.P., Garthe I., Geyer H. (2018). IOC consensus statement: Dietary supplements and the high-performance athlete. Int. J. Sport Nutr. Exerc. Metab..

[3] Kerksick C.M., Wilborn C.D., Roberts M.D., Smith-Ryan A., Kleiner S.M., Jäger R., Collins R., Cooke M., Davis J.N., Galvan E. (2018). ISSN exercise & sports nutrition review update: Research & recommendations. J. Int. Soc. Sports Nutr..

[4] Evans M., Tierney P., Gray N., Hawe G., Macken M., Egan B. (2018). Acute ingestion of caffeinated chewing gum improves repeated sprint performance of team sport athletes with low habitual caffeine consumption. Int. J. Sport Nutr. Exerc. Metab..

[5] Grgic J., Mikulic P., Schoenfeld B.J., Bishop D.J., Pedisic Z. (2019). The influence of caffeine supplementation on resistance exercise: A review. Sport Med..

[6] Warren G., Park N., Maresca R., Mckibans K., Millard-Stafford M. (2010). Effect of caffeine ingestion on muscular strength and endurance: A Meta-Analysis. Med. Sci. Sport Exerc..

[7] Graham T.E. (2001). Caffeine and exercise metabolism, endurance and performance. Sport Med..

[8] Todd A., Daniel W. (2010). Efficacy of caffeine ingestion for short-term high-intensity performance: A systematic review. J. Strength Cond. Res..

[9] Bishop D.J., Girard O. (2013). Determinants of team-sport performance: Implications for altitude training by team-sport athletes. Br. J. Sports Med..

[10] Mielgo-Ayuso J., Calleja-Gonzalez J., Del Coso J., Urdampilleta A., León-Guereño P., Fernández-Lázaro D. (2019). Caffeine supplementation and physical performance, muscle damage and perception of fatigue in soccer players: A systematic review. Nutrients.

[11] Salinero J.J., Lara B., Del Coso J. (2019). Effects of acute ingestion of caffeine on team sports performance: A systematic review and meta-analysis. Res Sport Med..

[12] McLellan T.M., Caldwell J.A., Lieberman H.R. (2016). A review of caffeine’s effects on cognitive, physical and occupational performance. Neurosci. Biobehav. Rev..

[13] Umemura T., Ueda K., Nishioka K., Hidaka T., Takemoto H., Nakamura S., Jitsuiki D., Soga J., Goto C., Chayama K. (2006). Effects of acute administration of caffeine on vascular function. Am. J. Cardiol..

[14] Ruíz-Moreno C., Lara B., Brito de Souza D., Gutiérrez-Hellín J., Romero-Moraleda B., Cuéllar-Rayo Á., Del Coso J. (2019). Acute caffeine intake increases muscle oxygen saturation during a maximal incremental exercise test. Br. J. Clin. Pharmacol..

[15] Broxterman R.M., Ade C.J., Craig J.C., Wilcox S.L., Schlup S.J., Barstow T.J. (2015). Influence of blood flow occlusion on muscle oxygenation characteristics and the parameters of the power-duration relationship. J. Appl. Physiol..

[16] Vanhatalo A., Fulford J., DiMenna F.J., Jones A.M. (2010). Influence of hyperoxia on muscle metabolic responses and the power-duration relationship during severe-intensity exercise in humans: A ^31^ P magnetic resonance spectroscopy study. Exp. Physiol..

[17] Polito M.D., Souza D.B., Casonatto J., Farinatti P. (2016). Acute effect of caffeine consumption on isotonic muscular strength and endurance: A systematic review and meta-analysis. Sci. Sports.

[18] Astorino T.A., Rohmann R.L., Firth K. (2008). Effect of caffeine ingestion on one-repetition maximum muscular strength. Eur. J. Appl. Physiol..

[19] Astorino T.A., Martin B.J., Schachtsiek L., Wong K., Ng K. (2011). Minimal effect of acute caffeine ingestion on intense resistance training performance. J. Strength Cond. Res..

[20] Duncan M., Oxford S. (2011). The effect of caffeine ingestion on mood state and bench press performance to failure. J. Strength Cond. Res..

[21] Hudson G.M., Green J.M., Bishop P.A., Richardson M.T. (2008). Effects of caffeine and aspirin on light resistance training performance, perceived exertion, and pain perception. J. Strength Cond. Res..

[22] Feito Y., Heinrich K., Butcher S., Poston W. (2018). High-intensity functional training (HIFT): Definition and research implications for improved fitness. Sports.

[23] Box A.G., Feito Y., Petruzzello S.J., Mangine G.T. (2018). Mood state changes accompanying the Crossfit Open ^TM^ competition in healthy adults. Sports.

[24] Jagim A.R., Rader O., Jones M.T., Oliver J.M. (2015). The physical demands of multi-modal training competitions and their relationship to measures of performance. J. Strength Cond. Res..

[25] Butcher S.J., Judd T.B., Benko C.R., Horvey K.J., Pshyk A.D. (2015). Relative intensity of two types of CrossFit exercise: Acute circuit and high-intensity interval exercise. J. Fit. Res..

[26] Fernández J.F., Solana R.S., Moya D., Marin J.M.S., Ramón M.M. (2015). Acute physiological responses during crossfit^®^ workouts. Eur. J. Hum. Mov..

[27] Butcher S.J., Neyedly T.J., Horvey K.J., Benko C.R. (2015). Do physiological measures predict selected CrossFit^®^ benchmark performance?. J. Sports Med..

[28] Crawford D.A., Drake N.B., Carper M.J., DeBlauw J., Heinrich K.M. (2018). Are changes in physical work capacity induced by high-intensity functional training related to changes in associated physiologic measures?. Sports.

[29] Rountree J.A., Krings B.M., Peterson T.J., Thigpen A.G., McAllister M.J., Holmes M.E., Smith J.W. (2017). Efficacy of carbohydrate ingestion on crossfit exercise performance. Sports.

[30] Fogaça L.J., Santos S.L., Soares R.C., Gentil P., Naves J.P., Dos W.S., Pimentel G.D., Bottaro M., Mota J.F. (2020). Effect of caffeine supplementation on exercise pndomized, erformance, power, markers of muscle damage, and perceived exertion in trained CrossFit men: A randomized double-blind, placebo-controlled crossover trial. J. Sports Med. Phys. Fit..

[31] Southward K., Rutherfurd-Markwick K.J., Ali A. (2018). The effect of acute caffeine ingestion on endurance performance: A systematic review and meta–analysis. Sport Med..

[32] Graham-Paulson T., Perret C., Goosey-Tolfrey V. (2016). Improvements in cycling but not handcycling 10 km time trial performance in habitual caffeine users. Nutrients.

[33] Pickering C., Kiely J. (2018). What should we do about habitual caffeine use in athletes?. Sport Med..

[34] O’Connor P.J., Motl R.W., Broglio S.P., Ely M.R. (2004). Dose-dependent effect of caffeine on reducing leg muscle pain during cycling exercise is unrelated to systolic blood pressure. Pain.

[35] Burke L.M. (2008). Caffeine and sports performance. Appl. Physiol. Nutr. Metab..

[36] Kamimori G.H., Karyekar C.S., Otterstetter R., Cox D.S., Balkin T.J., Belenky G.L., Eddington N.D. (2002). The rate of absorption and relative bioavailability of caffeine administered in chewing gum versus capsules to normal healthy volunteers. Int. J. Pharm..

[37] Kliszczewicz B., Snarr R.L., Esco M. (2014). Metabolic and cardiovascular response to the Crossfit workout “cindy”: A pilot study. J. Sport Hum. Perform..

[38] Borg G.A. (1982). Psychophysical bases of perceived exertion. Med. Sci. Sport Exerc..

[39] Mora-Rodríguez R., Pallarés J.G., López-Gullón J.M., López-Samanes Á., Fernández-Elías V.E., Ortega J.F. (2015). Improvements on neuromuscular performance with caffeine ingestion depend on the time-of-day. J. Sci. Med. Sport.

[40] Mora-Rodríguez R., Pallarés J.G., López-Samanes Á., Ortega J.F., Fernández-Elías V.E. (2012). Caffeine ingestion reverses the circadian rhythm effects on neuromuscular performance in highly resistance-trained men. PLoS ONE.

[41] Cohen J. (1988). Statistical Power Analysis for the Behavioral Sciencess.

[42] San Juan A.F., López-Samanes Á., Jodra P., Valenzuela P.L., Rueda J., Veiga-Herreros P., Pérez-López A., Domínguez R. (2019). caffeine supplementation improves anaerobic performance and neuromuscular efficiency and fatigue in Olympic-level boxers. Nutrients.

[43] Schneiker K.T., Bishop D., Dawson B., Hackett L.P. (2006). Effects of caffeine on prolonged intermittent- sprint ability in team-sport athletes. Med. Sci. Sport Exerc..

[44] Beck T.W., Housh T.J., Schmidt R.J., Johnson G.O., Housh D.J., Coburn J.W., Malek M.H. (2006). The acute effects of a caffeine-containing supplement on strength, muscular endurance, and anaerobic capabilities. J. Strength Cond. Res..

[45] Woolf K., Bidwell W.K., Carlson A.G. (2008). The effect of caffeine as an ergogenic aid in anaerobic exercise. Int. J. Sport Nutr. Exerc. Metab..

[46] Lane S.C., Hawley J.A., Desbrow B., Jones A.M., Blackwell J.R., Ross M.L., Zemski A.J., Burke L.M. (2013). Single and combined effects of beetroot juice and caffeine supplementation on cycling time trial performance. Appl. Physiol. Nutr. Metab..

[47] Da Silva V.L., Messias F.R., Zanchi N.E., Gerlinger-Romero F., Duncan M.J., Guimarães-Ferreira L. (2015). Effects of acute caffeine ingestion on resistance training performance and perceptual responses during repeated sets to failure Effects of acute caffeine ingestion on resistance training performance and perceptual responses during repeated sets. J. Sports Med. Phys. Fit..

[48] (2008). Glaister, M; Multiple-sprint work: methodological physiological, and experimental issues. Int. J. Sports Physiol..

[49] Guest N., Corey P., Vescovi J., El-Sohemy A. (2018). Caffeine, CYP1A2 genotype, and endurance performance in athletes. Med. Sci. Sports Exerc..

[50] Puente C., Abian-Vicen J., Del Coso J., Lara B., Salinero J.J. (2018). The CYP1A2-163C>A polymorphism does not alter the effects of caffeine on basketball performance. PLoS ONE.

[51] Ali A., O’Donnell J., Foskett A., Rutherfurd-Markwick K. (2016). The influence of caffeine ingestion on strength and power performance in female team-sport players. J. Int. Soc. Sports Nutr..

[52] Irwin B.C., Scorniaenchi J., Kerr N.L., Eisenmann J.C., Feltz D.L. (2012). Aerobic exercise is promoted when individual performance affects the group: A test of the kohler motivation gain effect. Ann. Behav. Med..

[53] Brupbacher G., Harder J., Faude O., Zahner L., Donath L. (2014). Music in CrossFit^®^—Influence on performance, physiological, and psychological parameters. Sports.

[54] Morton R.H. (2009). Deception by manipulating the clock calibration influences cycle ergometer endurance time in males. J. Sci. Med. Sport.

[55] Crawford D.A., Drake N.B., Carper M.J., DeBlauw J., Heinrich K.M. (2018). Validity, reliability, and application of the session-RPE method for quantifying training loads during high intensity functional training. Sports.

[56] Saunders B., de Oliveira L.F., da Silva R.P., de Salles Painelli V., Gonçalves L.S., Yamaguchi G., Mutti T., Maciel E., Roschel H., Artioli G.G. (2017). Placebo in sports nutrition: A proof-of-principle study involving caffeine supplementation. Scand. J. Med. Sci. Sport.

